# Ameloblastoma in the mandible

**DOI:** 10.1016/S1808-8694(15)30589-9

**Published:** 2015-10-19

**Authors:** Marcelo Medeiros, Gabriela Granja Porto, Jose Rodrigues Laureano Filho, Luís Portela, Ricardo Holanda Vasconcellos

**Affiliations:** 1 Specialist in maxillo-facial surgery and traumatology, Master's program in maxillo-facial surgery and traumatology student; 2 Specialist in maxillo-facial surgery and traumatology, MSc student - MFS FOP-UPE.; 3 PhD in maxillo-facial surgery and traumatology, adjunct professor of maxillo-facial surgery and traumatology – University of Pernambuco.; 4 MSc in Biomedical Engineering, Assistant Professor of Tooth Implants at the University of Pernambuco.; 5 PhD in Maxillo-facial Surgery and Traumatology, Adjunct Professor of Maxillo-facial Surgery and traumatology – University of Pernambuco.

**Keywords:** ameloblastoma, surgery, mandible patology

## INTRODUCTION

The ameloblastoma is an enamel tissue tumor, which does not differentiate to form the enamel. It is benign and of ectodermic origin. Although considered a benign tumor, its clinical behavior can be considered of middle ground, between benign and malignant. The tumor is characterized by slow but persistent growth and infiltration in adjacent tissue^1^, ^2^.

## CASE PRESENTATION

**Figure 1:**
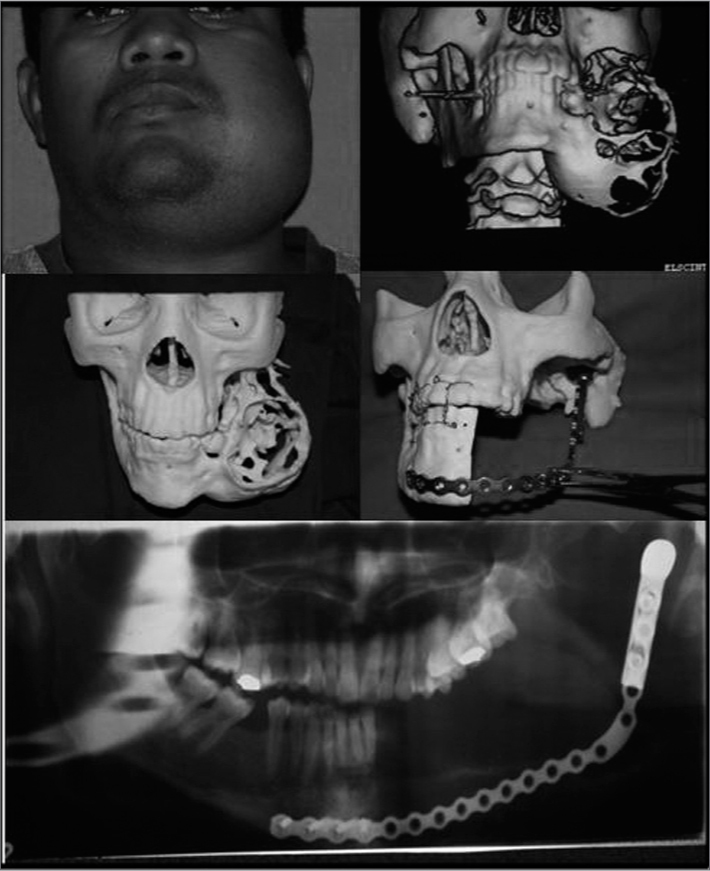
Postoperative clinical and radiographic aspects, prototyping model and postoperative radiographic aspect.

R.T.B., male, 30 years old, came to the Department of Maxillo-Facial Surgery complaining of a bulging in his left-side mandible, which had been enlarging for the past ten years. An incisional biopsy was carried out through the oral cavity. The pathology finding was ameloblastoma. In order to better plan access to the lesion and mandible reconstruction after tumor exeresis, we made a model through the prototyping technique. Treatment chose was hemimandibulectomy, with mandibular condyle loosening on the ipsilateral side, since the tumor had already invaded the cortical bone, with immediate reconstruction with reconstruction plate and condyle coupled to it. Currently the patient has been under observation for four years, without signs of recurrence (Figure 1).

## DISCUSSION

Ameloblastomas are classified in unicystic, solid or multicystic, peripheral and malignant subtypes in conventional or multicystic solid (86% of the cases), unicystic (13% of the cases) and peripheral or extra-osseous (about 1% of the cases)^3^. Such distinction is important because the treatment of a unicystic lesion can be more conservative, for it has a less aggressive behavior and smaller size than its solid or multicystic counterpart^4^.

Typical ameloblastoma starts insidiously as a central bony lesion which is slowly destructive; however tends to expand the bone instead of punching a hole through it. The tumor is rarely painful, unless infected and usually does not cause signs and symptoms of nerve involvement, even when large^3^.

Radiographically, the most common aspect of multicystic ameloblastomas is a multilocular lesion; often times described as having the appearance of “soap bubbles” when large, and are described as honeycomb when small. There is frequently a lingual cortical and oral expansion, and usually the teeth roots adjacent to the tumor are resorbed. Unicystic ameloblastomas present a radiolucent image that surrounds the crown of an unerupted tooth or, they simply appear as a well defined radiotransparent areas^3^.

Gender distribution as far as ameloblastomas are concerned is 1:14,5. The age of most frequent onset is the 3rd and 4th decades of life^6^. The mandible is about four times more affected than the maxilla^6^. About ¾ of mandible tumors are located in the mandible ramus and molar teeth. When it involves the maxilla, the posterior region is also the one most affected, and as it develops, the tumor may involve the maxillary sinus and the orbit^5^.

There is a trend to treat unicystic ameloblastomas by curettage with 10%-15% of recurrence; however, avoiding patient mutilation. In the solid or multicystic tumor, it is necessary to have radical surgical excision, with resection of the affected bone with at least 15 mm of healthy tissue as safety margin^6^. The mucosa in contact with the tumor must be entirely removed, because it may contain ameloblastic cells that can contaminate the graft during reconstruction^1^.

It is important to stress the capacity the tumor has to develop late recurrences. Because of its slow growth, these recurrences may take years, and even decades to happen after the first surgery^1^.

## FINAL COMMENTS

Thus, we conclude that radical surgery is the treatment of choice most of the times. We must stress that radiographic methods are not able to determine the exact disease extension and the recurrence rate is close to 100% when not treated properly.
